# A Biased Median Filtering Algorithm for Segmentation of Intestinal Cell Gland Images

**DOI:** 10.1100/tsw.2006.41

**Published:** 2006-02-22

**Authors:** Hai-Shan Wu, Joan Gil

**Affiliations:** Department of Pathology, Box 1194, Mount Sinai School of Medicine, One Gustave L. Levy Place, New York, NY 10029, USA

**Keywords:** cell image, image segmentation, cell gland, intestine cell

## Abstract

In this paper, we introduce a biased median filtering image segmentation algorithm for intestinal cell glands consisting of goblet cells. While segmentation of individual cells are generally based on the dissimilarities in intensities, textures, and shapes between cell regions and background, the proposed segmentation algorithm of intestine cell glands is based on the differences in cell distributions. The intestine cell glands consist of goblet cells that are distributed in the chain-organized patterns in contrast to the more randomly distributed nongoblet cells scattered in the bright background. Four biased median filters with long rectangular windows of identical dimension, but different orientations, are designed based on the shapes and distributions of cells. Each biased median filter identifies a part of gland segments in a particular direction. The complete gland regions are the combined responses of the four biased median filters. A postprocessing procedure is designed to reduce the defects that may occur when glands are located very close together and to narrow the gapping areas because of the thin distribution of goblet cells. Segmentation results of real intestinal cell gland images are provided to show the effectiveness of the proposed algorithm.

## INTRODUCTION

In pathology, diagnoses of diseases are based on the recognition of visual clues or diagnostic criteria from various tissue specimens by a trained observer. A diagnosis made by a pathologist is often based on the subjective estimation and comparison of parameters such as the size of nuclei, nucleoli and chromatin clumps, roughness of chromatin appearance, smoothness and roundness of nuclear shapes, etc.[1,2,3,4,5,6,7,8,9]. Algorithms for identification and segmentation of single cells or nuclei have been developed based on the dissimilarities between the contents inside and outside of the individual cells or nuclei[10,11,12,13,14,15]. Although individual cells provide important diagnosis parameters, collective cells that form particular structured clusters may also reveal useful information for diagnosis[16]. In the case of small intestines that are generally fixed with formalin and embedded in paraffin, the microscopic images, generated by sectioning the paraffinized tissues into 4 µm-thin slices, mounted onto glass slides, and stained with H&E (Hematoxylin and Eosin), show the round and roughly oblong small intestinal cell glands or crypts[17,18]. These cell glands mainly consist of mucin-containing columnar cells or goblet cells that are evenly distributed and arranged in parallel at the base. The size and shape of those cell glands can change on injury. Under ischemia, intestinal cell glands become atrophic or microcystic, whereas in idiopathic inflammatory bowel disease, they usually show severe distortion including crypt branching and irregularity. In addition to the goblet cells in the glands, a diverse population of endocrine cells is scattered among the epithelial cells lining the intestinal villi, small and large intestinal crypts. To evaluate quantitatively the size, shape, and other information of the intestine cell glands, the gland regions must be identified and segmented from the background scattered with the disturbing nongoblet cells.

## ALGORITHM

In microscopic images, the intestinal cell glands that consist of goblet cells show roughly long elliptical shapes while the background regions are scattered randomly with the endocrine and epithelial cells that are generally smaller and roughly round as shown in Fig. 1 (A). Both goblet cells and the group of endocrine and epithelial cells are dark in intensities against the bright background. The significant intensity difference between the dark cell and bright background pixels makes the cell separation relatively easy by a simple thresholding. To separate the cell gland from the endocrine and epithelial cells, we still need to further discriminate the goblet cells from the other kinds of cells that are always present in the intestinal cell gland images.

The goblet cells that form the cell glands generally sit side by side in a chain structure while the nongoblet cells randomly scattered apart from each other. Since there is little difference in intensities between the two groups of cells, we develop the algorithm based on the binary images without considering the variation in intensities to simplify the computations and effects of the intensity fluctuations. From the sample image in Fig. 1, it is observed that the goblet cells are generally larger and more closely located in a chain structure. If we use a window large enough to enclose several goblet cells in a row, we expect that number of black pixels inside the window will be likely larger for pixels inside a cell gland than those outside. If a parameter, $\gamma$, is defined as the ratio of number of black pixels inside a window to the total area of the window, the ratio $\gamma$ is likely bigger for goblet cell pixels that nongoblet ones. To produce the largest difference in $\gamma$ between goblet and nongoblet cells, the shape and size of windows should be adaptive to the size and distributions of cells in the cell image.

Suppose the nongoblet cells can be approximated in shape by circles while goblet cells approximated by long ellipses. Let the radius of a nongoblet cell be $b$. Define the short and long axes of a goblet ellipse to be $2a$ and $2\alpha a$, respectively, where $\alpha$ is the scale or ratio between the two elliptical axes. The goblet cells are located horizontally in parallel as shown in Fig. 2 (A) where the gap between two neighboring goblet cells is approximated by $d_a$. Let the window be a rectangle of size $(2x_0) \times (2y_0)$ and its center be located at the origin. Although $x_0$ should be as large as possible to enclose more goblet cells, its value is limited by the curves of the glands. Let $x_0$ is such as value that either $M_a$ goblet cells or $M_b$ nongoblet cells are covered by the window in the $x$ axis. The ratio for goblet cells (ratio of the total number of pixels in the cells in window to the total area of the window) is found to be

$$\gamma_a(y_0) = \frac{M_a}{2\alpha y_0 x_0}\left( y_0\sqrt{(\alpha a)^2 - y_0^2} + (\alpha a)^2 \sin^{-1}\frac{y_0}{\alpha a}\right) \text{ if } y_0 \le \alpha a \text{, and } \gamma_a(y_0) = \frac{M_a \alpha a^2 \pi}{4 y_0 x_0} \text{ if } y_0 > \alpha a,$$

where $M_a$ is the number of goblet cells in the window. Similarly, the nongoblet cells outside the cell gland regions are modeled as circles along the horizontal line, but in a larger distance, $d_b$, from each other as shown in Fig. 2 (B). The ratio for the nongoblet cells is evaluated as

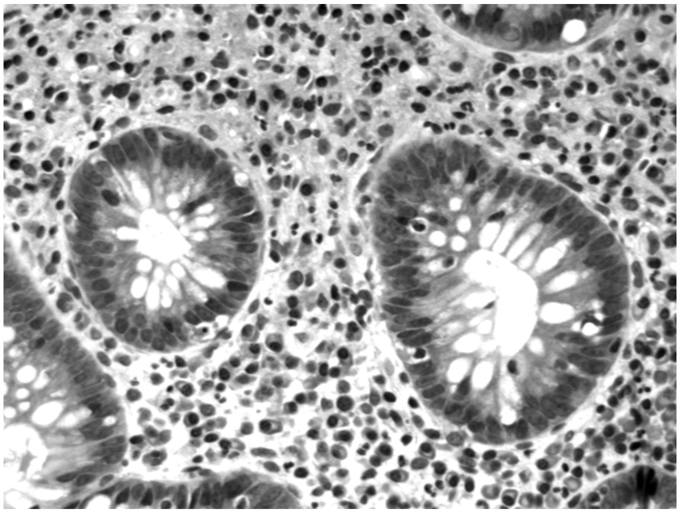

A

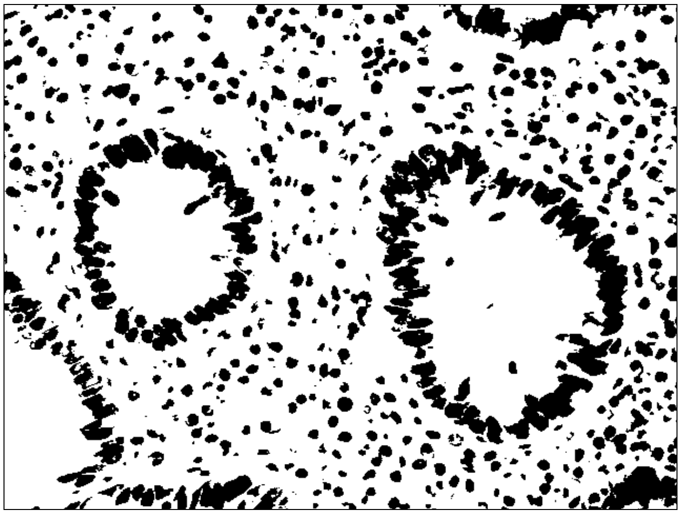

B

**FIGURE 1**. (A) A typical image of small intestine glands. There are two complete glands in the middle and four partial ones at the image borders. (B) A threshold segmentation separating the regions of cells in both groups from the background.

$$\gamma_b(y_0) = \frac{M_b}{2y_0x_0}\left(y_0\sqrt{b^2 - y_0^2} + b^2\sin^{-1}\frac{y_0}{b}\right) \text{ if } y_0 \le a \text{, and } \gamma_b(y_0) = \frac{M_b b^2\pi}{4y_0x_0} \text{ if } y_0 > a,$$

where $M_b$ is the number of the circular nongoblet cells in the window. The value $y_0$, half of the window side in the vertical direction, should be selected such that $\gamma_a(y_0) - \gamma_b(y_0)$, the ratio difference of the goblet and nongoblet cells, is maximized.

To find the best window dimension for the typical intestinal cell gland images, we set the ideal image model in Fig. 2 based on the segmented binary image in Fig. 1 (B). Unlike photographic images from the world of real nature that vary significantly from images to images, the microscopic intestine cell images appear actually very similar. Models developed on typical cell images may be applicable to other cell images provided the imaging settings such as the staining and magnifications are identical.

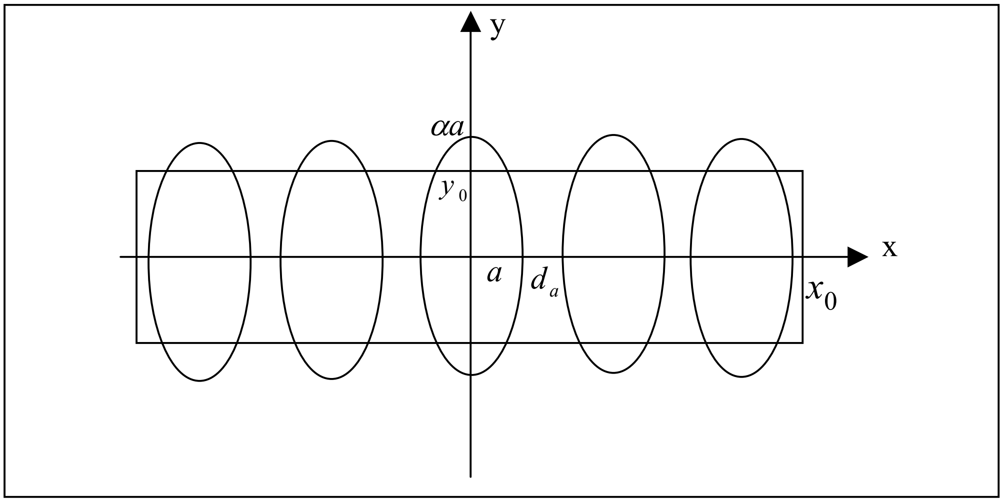

**A**

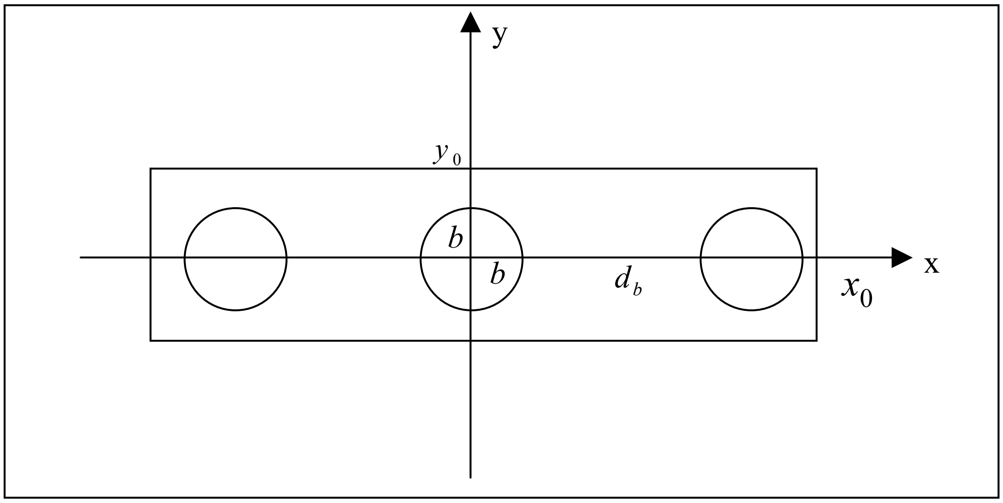

**B**

**FIGURE 2.** The simulations of gland and background regions. (A) Goblet cells in a row forming a piece of segment of a gland band. The shape of goblet cells are elliptical with long axis roughly trice as long as the short one. The cells sit well in parallel. (B) Nongoblet (endocrine or epithelial) cells loosely scattered in the background. The endocrine and epithelial cells have nearly round shape and sit loosely in distance from each other.

To set the appropriate parameter values, we need to estimate the average size of goblet cell nuclei. Since the cells of the same type usually have the similar cell size, the histograms of the number of regions against the region size will provide a useful function that tends to have similarly sized regions in narrow clusters. The goblet cell nuclei are generally sitting side by side in chains and often attached, while the nongoblet cells are more isolated and sparsely scattered as shown in Fig. 1 (B). To estimate the average size of the goblet cell nuclei, we need to segment and identify the typical goblet cell nuclei from background and other cell nuclei. Since goblet cell nuclei are often attached after thresholding, we apply a morphological erosion operation intended to separate the attached goblet cell regions. Fig. 3 (A) shows an erosion of image in Fig. 1 (B) by a round structuring element of radius of three pixels. To restore the nuclear size, a reverse conditional dilation of the same structuring element is applied obtaining the image shown in Fig. 3 (B). The expansion is conditionally grown in the regions of the image in Fig. 1 (B) to avoid

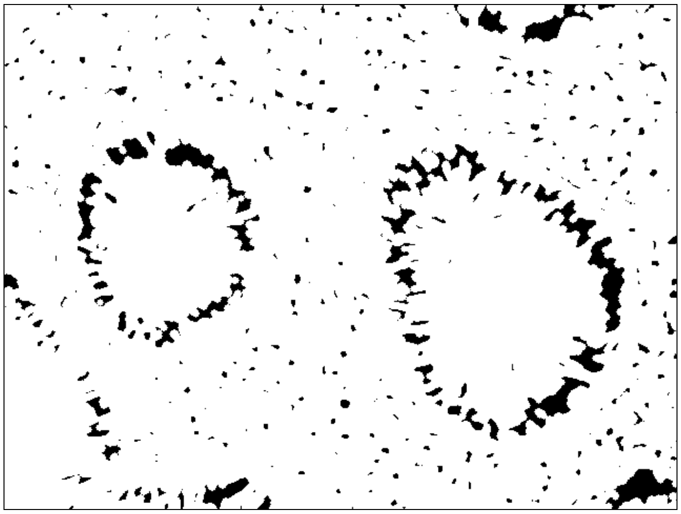

**A**

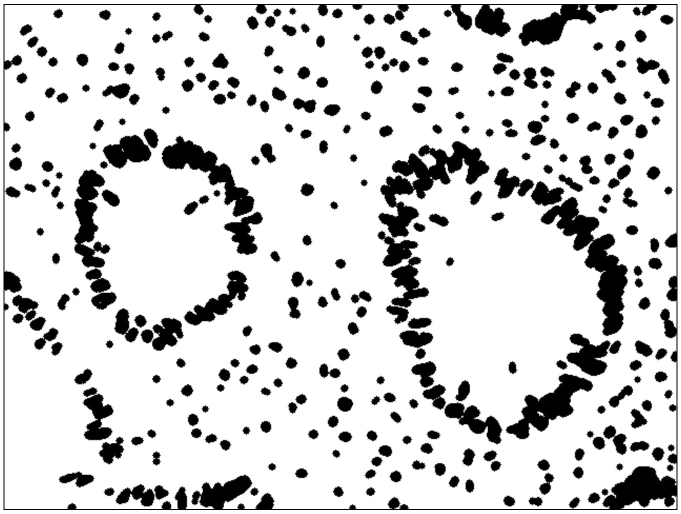

**B**

**FIGURE 3.** Morphological operations and histograms. (A) Erosion of image in Fig. 1 (B) by a round structure element of radius of three pixels; (B) conditional dilation of image in (A) by a round structure element of radius of three; (C) histogram of size of detached regions; (D) histogram of number of regions vs. region size; (E) histogram in (D) smoothed by a low-pass filter.

possible overgrowing. If we measure the size of each black region, we get the histogram of regional size as shown in Fig. 3 (C). There are totally 412 separate regions. Shown in Fig. 3 (D) is the histogram of number of regions against the regional size. High value in the histogram implies larger number of regions having the same size. Since there are a large number of nongoblet cell nuclei that appear isolated, small and similar in size, the lower portion of the histogram corresponds to the nongoblet cell regions. It is easier to identify when the histogram is smoothed by a low-pass filter as shown in Fig. 3 (E). The curve drops to the low value in the range between 140 and 150. If we eliminate the regions whose size is smaller than 140, we get the regions in Fig. 4 (A) where vast majorities of nongoblet cells are removed. Some large regions corresponding to large spikes as high as over 2000 in Fig. 3 (C) are actually attached goblet cells. Fig. 3 (D) shows only partial histogram of regional numbers vs. size up to 500. There are

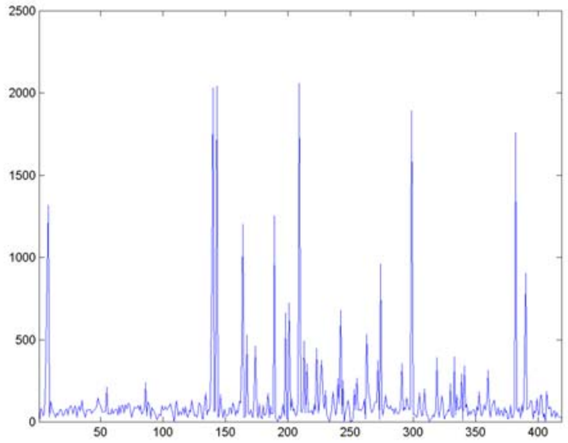

**FIGURE 3C**

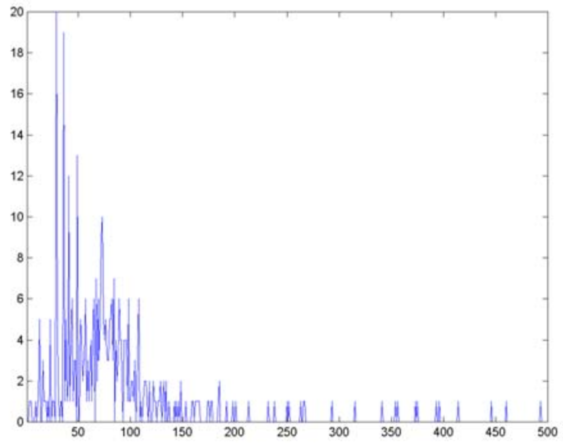

**FIGURE 3D**

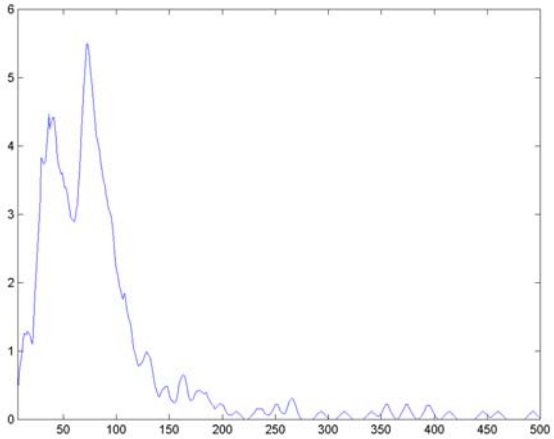

**FIGURE 3E**

very few nonzeroes beyond 500 horizontally in size in Fig. 3 (D). From the smoothed curve in Fig. 3 (E), the end of the high wave at the range between 140 and 150 means the separation point between the goblet and nongoblet cells. The size of 300, double of this point at 150, should be considered as regions with two or more goblet cells. Retaining the nuclear regions of size between 140 and 300, we obtain the image shown in Fig. 4 (B) where regions are considered as representative of isolated individual goblet cell nuclei. The average size of them will be considered as the estimation of the average size of goblet cell nuclei. The average size in this example is estimated as approximately 192. Fig. 5 shows another estimation of average size of goblet cell nuclei. Fig. 5 (E) displays the extracted isolated goblet nuclei from the gland chains with the estimated average nucleus size of 193. Two more testing images are shown in Fig. 6 where the estimated two average nucleus sizes are 181 and 195, respectively. The axis parameter is set

$$a = \sqrt{\frac{192}{\pi \alpha}}\,.$$

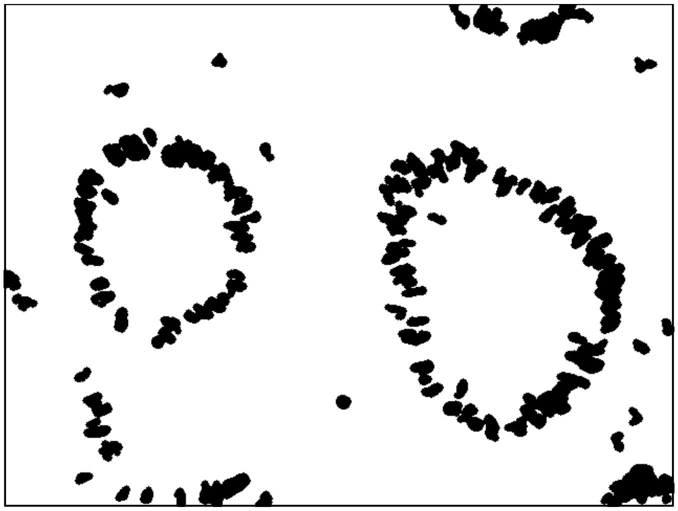

A

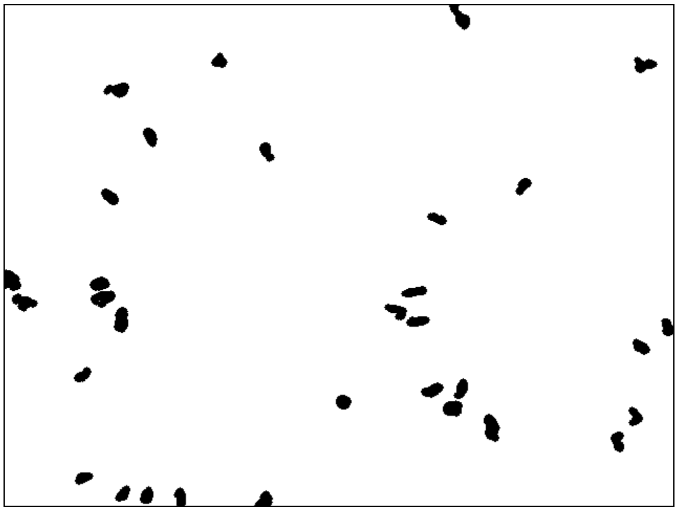

B

**FIGURE 4.** Extraction of detached regions as representative of average goblet cell nuclei in size. (A) Small-sized nongoblet cells removed first; (B) retaining the isolated single cells after the attached cells being removed from (A).

If $\alpha = 2$ when the long axis is about twice the short axis, we have the parameter $a = 5.528$. The elliptical shape of goblet cells has a minor axis similar to the diameter of the circular nongoblet cells. Thus, we set $b = a = 5.528$. The gaps between neighboring goblet cells in the chains range of zero to five. We simply set $d_a = 2$. Let the window cover five whole goblet cells horizontally because of the curves of the cell chains or $M_a = 5$. The nongoblet cells are generally located far from each other while adjacent goblet cells sit closer each other. The distance between neighboring nongoblet nuclei is approximately the size of the diameter of the nongoblet cells themselves. Thus, $d_b = 2b + d_a$. There are three nongoblet cells horizontally in the window or $M_b = 3$. We have

$$x_0 = \frac{M_a}{2}(2a + d_a) \cdot$$

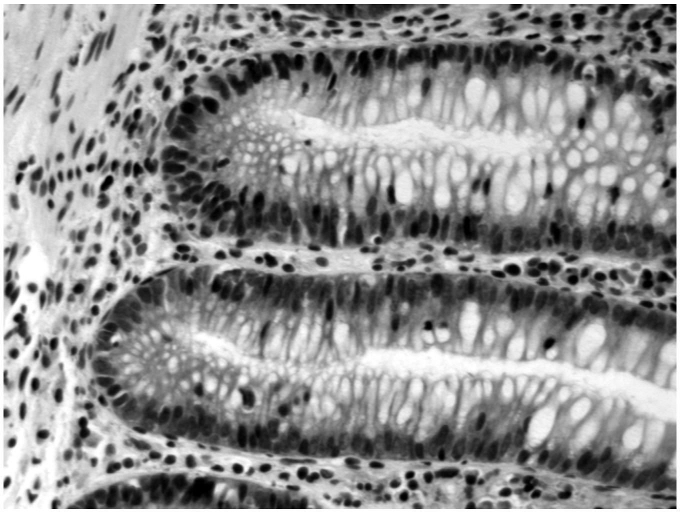

A

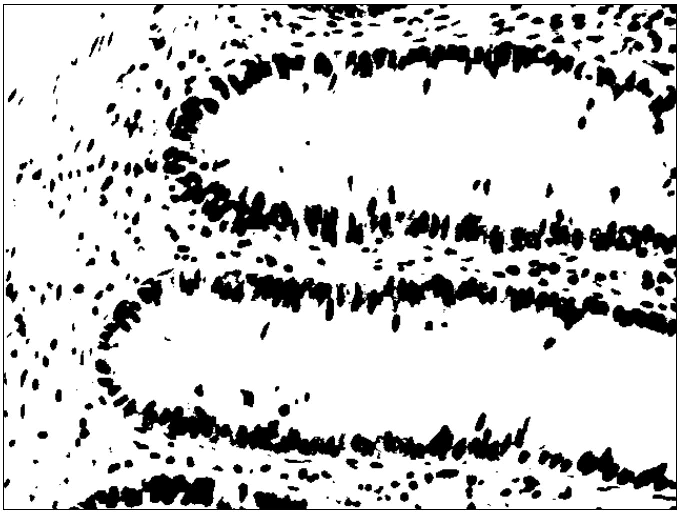

B

**FIGURE 5**. Extraction of detached goblet nucleus regions from another nuclear image. (A) Another original intestine gland image; (B) threshold-segmented; (C) erosion of image in (B) by a round structure element of radius of three pixels; (D) conditional dilation of image in (A) by a round structure element of radius of three; (E) retaining the isolated single cells after the attached cells being removed from (D).

Fig. 7 (A) displays $\gamma_a(y_0)$, the ratio of the goblet cell pixels to the total in the window against $y_0$, the half of the window dimension in $y$ axis, while Fig. 7 (B) shows $\gamma_b(y_0)$, the ratio of the nongoblet cell pixels to the total in the window against $y_0$. Fig. 7 (C) shows the ratio difference between $\gamma_a(y_0)$ and $\gamma_b(y_0)$. It is observed in Fig. 7 (C) that the highest ratio difference occurs near $y_0 = 8.5$, slightly lower than $\alpha a = 11.5$, the half of the major diameter. Thus, the window dimension in the $y$ axis should be set to be 8.5 so that the goblet and nongoblet cells are most separable. The window is therefore a rectangle of dimension 60 × 16 covering the five goblet cells horizontally and 80% of one goblet cell vertically. The resulting ratio matrix is then segmented with the threshold of

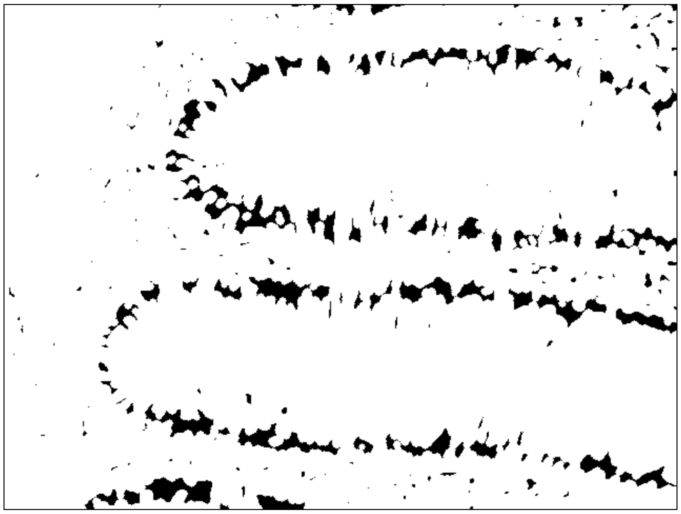

FIGURE 5C

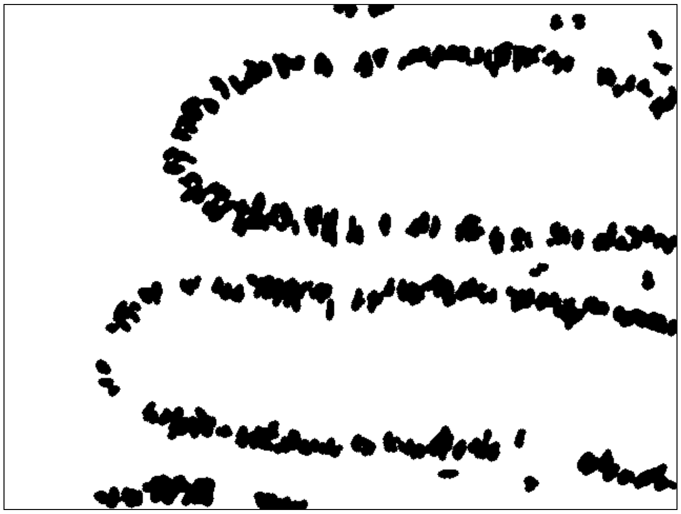

FIGURE 5D

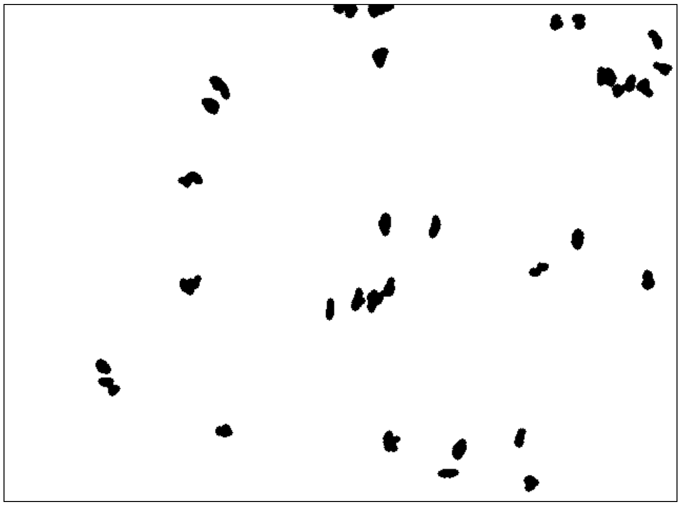

FIGURE 5E

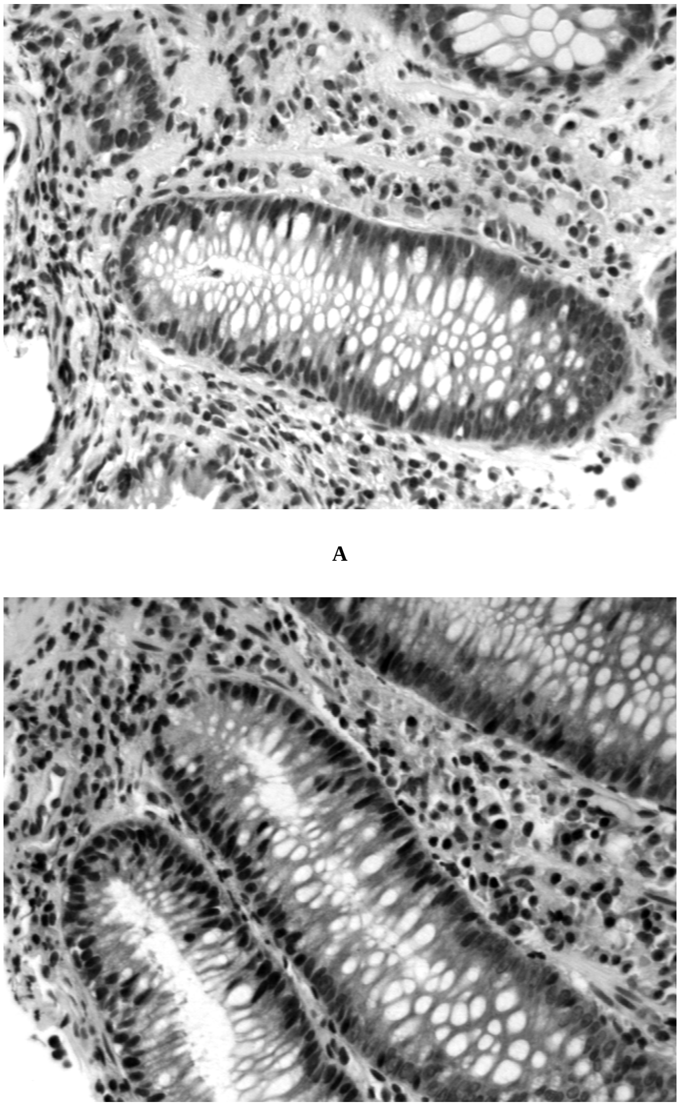

A

B

**FIGURE 6.** Two additional intestinal cell gland images.

$$\frac{\gamma_a(y_0)+\gamma_b(y_0)}{2},$$

the average ratio for goblet and nongoblet cells, at y0 = 8.5. We have the threshold

$$\frac{\gamma_a(8.5)+\gamma_b(8.5)}{2} \approx 0.48 .$$

Thus, we select the value at 48% if sorted in increasing order as the output comparing to that at 50% or the middle value of a normal median filter. A bias is dependent on the size, shape, and distributions of cells of both groups. If the cells in one or both groups are distributed more sparsely, the ratio threshold will be lower resulting in a larger downward bias to the filters.

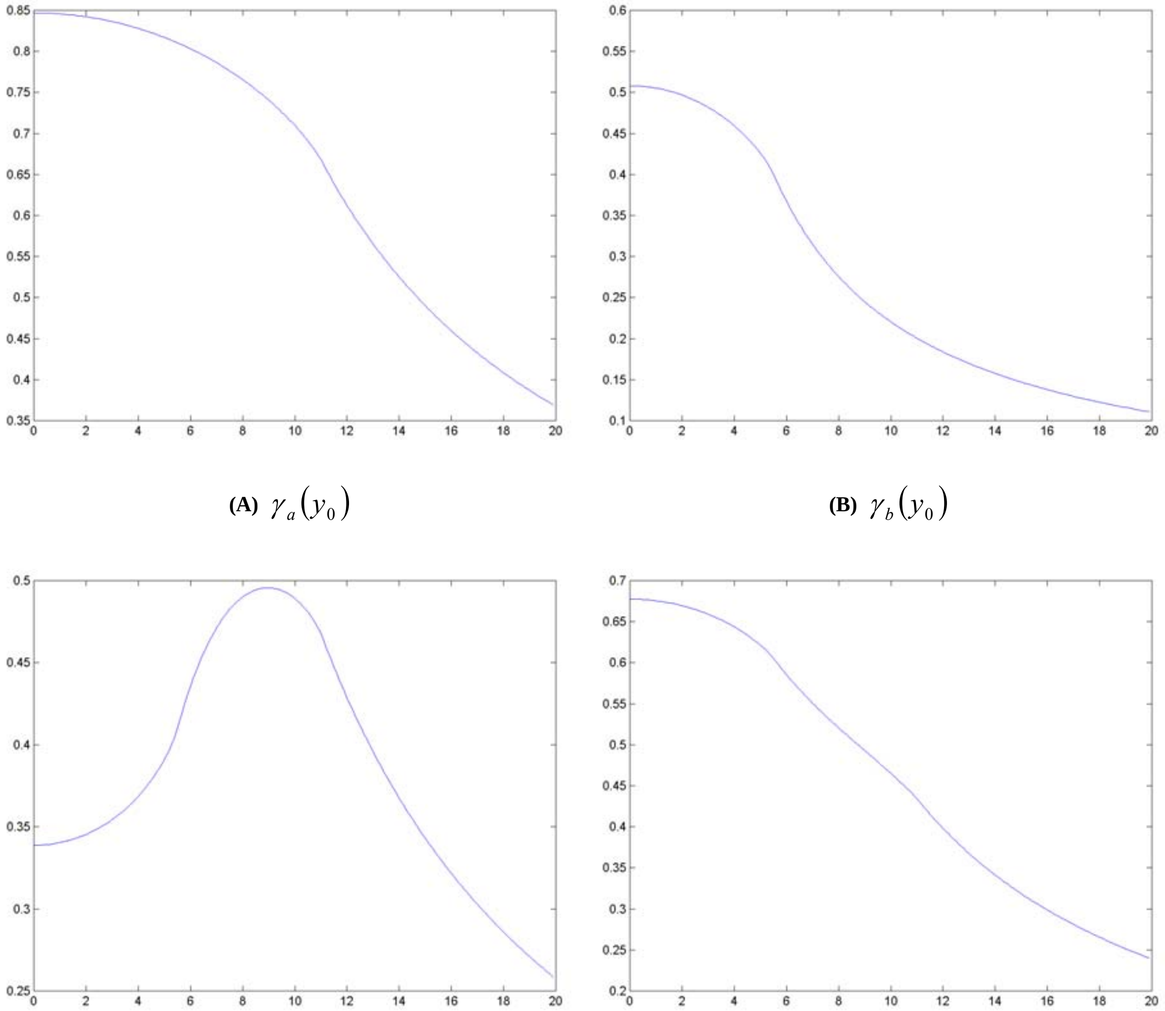

**(A)** $\gamma_a(y_0)$ **(B)** $\gamma_b(y_0)$

**(C)** $\gamma_a(y_0)-\gamma_b(y_0)$ **(D)** $\frac{1}{2}(\gamma_a(y_0)+\gamma_b(y_0))$

**FIGURE 7.** The ratios of number of cell pixels to the total in the window. (A) Ratio for goblet cells; (B) ratio for nongoblet cells; (C) the ratio difference between goblet and nongoblet cells; and (D) the average ratio of goblet and nongoblet cells.

We should note that the above biased median filter considers only the goblet cells in a chain segment lying horizontally. To obtain the regions of goblet cells in vertical chain segment, we apply the same biased median filter but with an extra rotation of

$$\frac{\pi}{2}$$

degree to the window. Similarly, we need another two biased median filters with the windows rotated by

$$\frac{\pi}{4} \text{ and } \frac{3\pi}{4}$$

degrees from the first one, respectively, to segment the cell gland segments that have slopes with the two coordinate axes. Combining all the segmented goblet cell regions from the four biased median filters produces the final cell gland segmentation.

In the cases where goblet nuclei are thinly distributed or glands are located very close to each other, a postprocessing procedure can be applied to eliminate most errors and improve the segmentation. In each of the outputs of the four different directional filters, the segmented gland regions have lengthened shapes fitting in rectangles with long sides pointing to the same directions as the filtering windows. However, many misclassified nongoblet nucleus regions, especially those located between two adjacent glands that are very close, the long sides of the rectangles may not be significantly larger than the short sides. Those misclassified regions or defects may then be eliminated based on the ratios between the long and short sides. On the other hand, the broken glands due to the uneven distributions of goblet cells or the sharp curves of the goblet cell chains at the heads of some glands may be repaired by morphological operations. A very long and narrow rectangle window with an assigned direction can be used to dilate and determine the gapping regions where the bias of the modified median filtering can be lowered allowing more pixels to be classified as the goblet nucleus pixels in the gapping area.

## RESULTS AND DISCUSSIONS

The original cell image, size of 640 × 480 and 256 levels, of intestinal cell glands in Fig. 1 (A) is acquired by a microscope with magnification of ×20 on H&E-stained glass slides of paraffinized tissues sectioned into a four micron-thin slice. Shown in Fig. 1 (B) is the segmented binary image by a simple intensity thresholding method. As described in the previous section, the windows are rectangles of size of 60 × 16 and the ratio threshold is 0.48. Figs. 8 (A–D) show the extracted cell gland regions in black by the four windows with the same size but rotations in angles of

$$0, \frac{\pi}{4} \text{ and } \frac{\pi}{2} \text{ and } \frac{3\pi}{4},$$

respectively. We can see obviously that each image in Figs. 8 (A–D) contain pieces of nuclear gland regions in a particular direction. Fig. 8 (E) shows the final cell gland regions by combining all the four partial cell gland regions in Figs. 8 (A–D). Comparing visually the segmented cell glands in Fig. 8 (E) to the original image in Fig. 1, the resulting segmented cell glands match the original well except occasional defects at the cell gland borders that may be reduced by morphological open operations. For visual comparison, Fig. 8 (F) shows the segmentation by a Gaussian filter with $\sigma^2 = 20^2$ followed by a manually adjusted thresholding. It is observed that the large unwanted nongoblet cell regions are present and the goblet cell regions that are supposed to be continuous are broken with large gaps.

Figs. 9–11 show the results of another intestinal cell gland image. The original image is shown in Fig. 5 (A). Fig. 9 shows the outputs of the four different directional biased median filters. In the original image in Fig. 5 (A), the two neighboring cell glands in the middle of image are located very close to each other. When the gaps between the neighboring cell glands are significantly smaller than the long side of the filter windows, the resulting cell gland regions in the gaps may be over-segmented because of the effects of the long windows covering extra goblet cells in the other unwanted cell glands. In addition, because of the specimen section cutting along the glands, the glands appear one-ended. The sharp turns at the head of each gland may cause the region under-segmented because of the insufficient number of goblet nuclei fitting inside the long filtering windows. The image in Fig. 9 (A) has a large portion of segmented gland lines since the lines are mostly parallel to the corresponding window used for the image in (A). The set union of the black pixels in the four images is shown in Fig. 10 (C) where defects occur between the two major glands and broken gaps appear in the goblet nuclear lines. Each of the four biased median filters consists of two steps, one to determine the ratio of the goblet nucleus pixels in a given window to the total

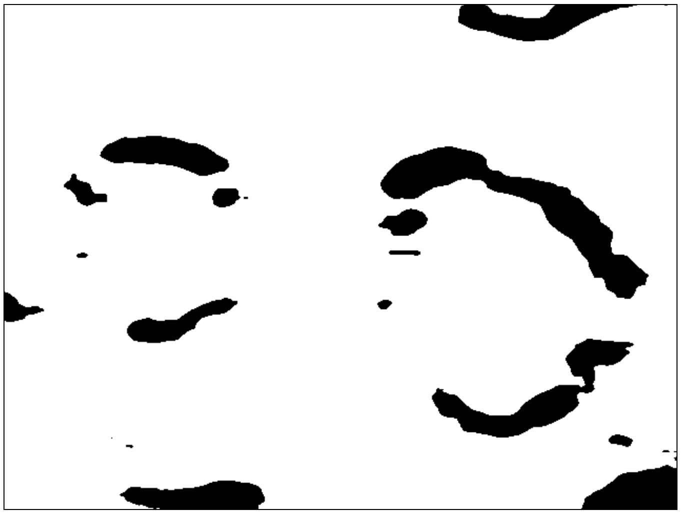

A

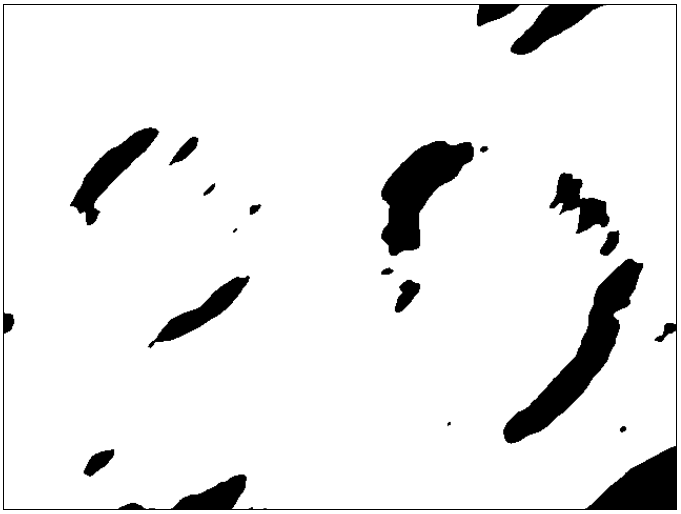

B

**FIGURE 8.** (A–D) The segmented gland regions by the four biased median filters with the same window size, but in different rotations as described in the text; (E) the combined segmentation by summing up all the black regions in the four images in (A–D); (F) segmentation with Gaussian filtering.

number of elements in the window followed by another comparison of the ratio to the bias threshold. The complete segmentation as shown in Fig. 10 (C) is the set union of the segmented goblet nucleus regions from the four separate biased median filters. This procedure is equivalent to first obtaining the combined ratio function as shown in Fig. 10 (A) by selecting the largest value among the four ratios of black pixels covered by the respective moving windows to the window size and then followed by a bias thresholding. Comparing with the Gaussian filtering in Fig. 10 (B) based on which the thresholding segmentation is obtained in Fig. 10 (D), the ratio image in (A) shows clearer and smoother gland lines. Fig. 11 (A) shows the five-level map for the four directional windowed filters, in which a nonblack level corresponds to the window that produces the largest ratio among the four. The shapes of regions are basically stretched out in the direction of its window. When fitting a region in the map into the smallest rectangle with the rotation angle the same as the corresponding window, we expect that the side of the rectangle in the

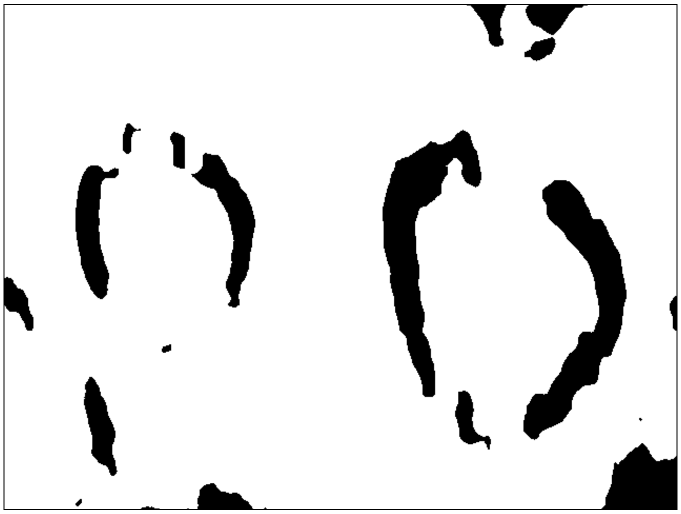

**FIGURE 8C**

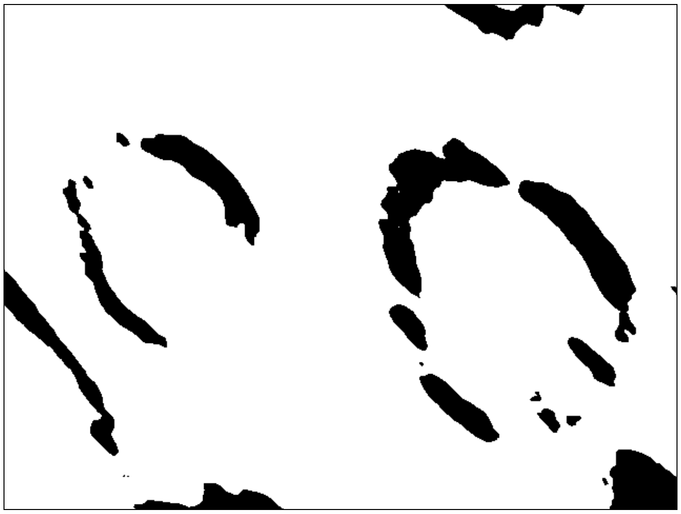

**FIGURE 8D**

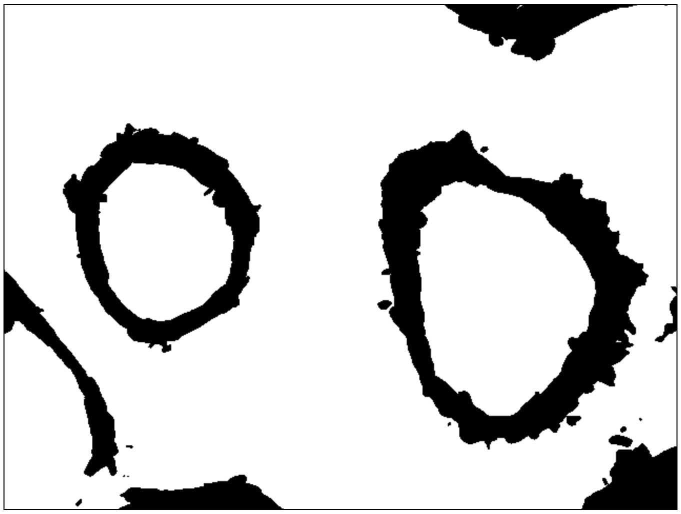

**FIGURE 8E**

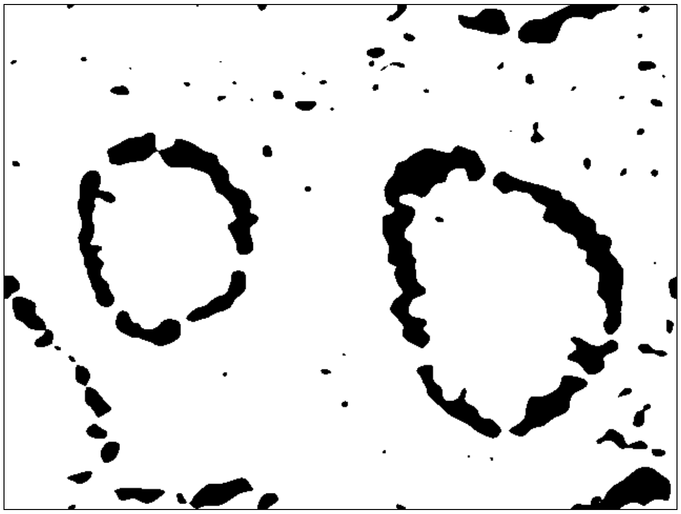

**FIGURE 8F**

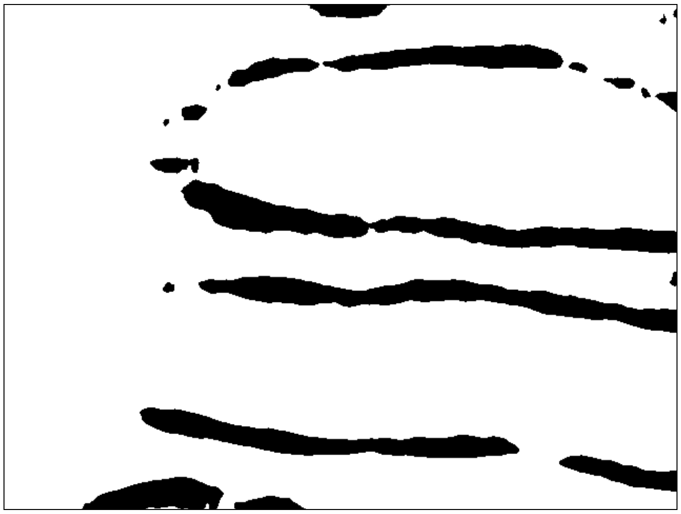

**A**

**FIGURE 9**. (A–D) The segmented gland regions by the four biased median filters with the same window size in rotations of degrees as described in the text.

window direction is much longer than the other side. If the side of the fitting rectangle of a given region in the window direction is 1.5 times longer than the other side and the size of the region is relatively large, the region is retained, otherwise the region is eliminated. Fig. 11 (B) shows the modified mapping after the regions in (A) either too small or unfit in shapes are removed. Fig. 11 (C) displays the possible expanding areas using morphological dilations with a slim rectangular structuring element of size $51 \times 3$ rotated accordingly to the directions of the respective windows. In order to allow more pixels in the extending area (black intensity) in Fig. 11 (C) to be classified into gland area, the bias is lowered in these regions by 0.1 and the newly added regions are shown in Fig. 11 (D). It is seen that thick areas occur in the gapping regions while thin lines appear elsewhere. The final segmentation is shown in Fig. 11 (E).

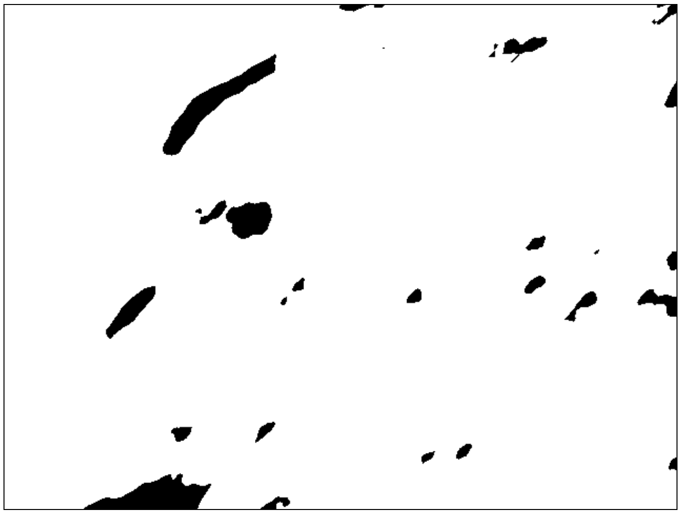

**FIGURE 9B**

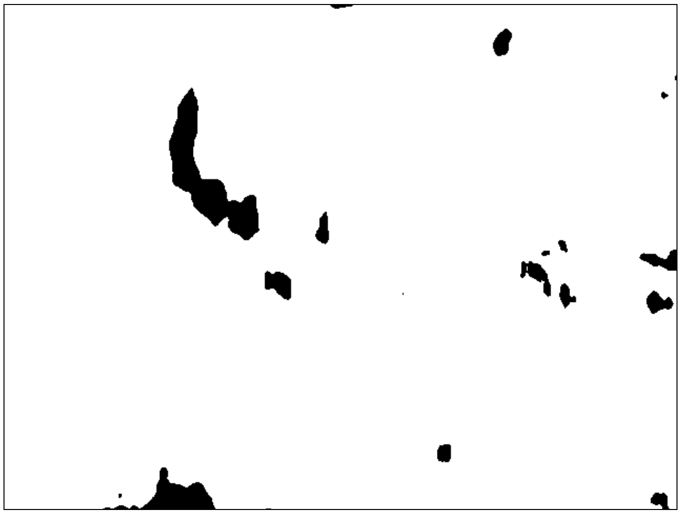

**FIGURE 9C**

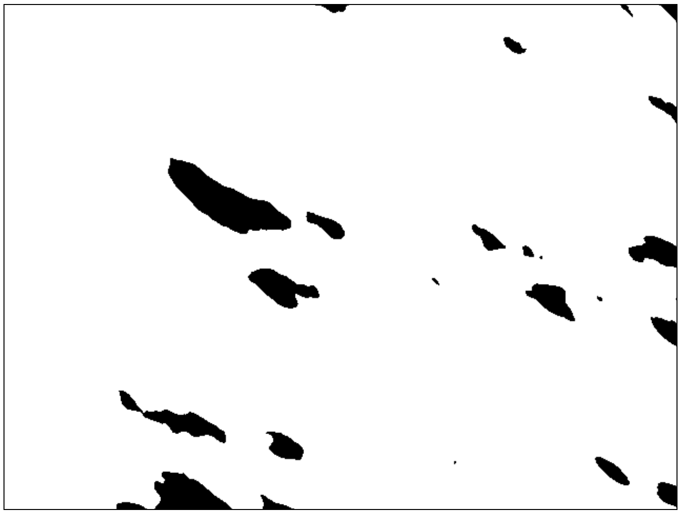

**FIGURE 9D**

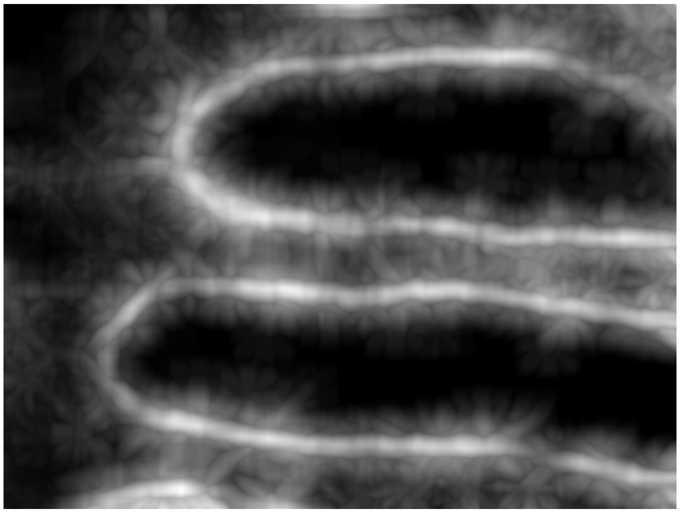

A

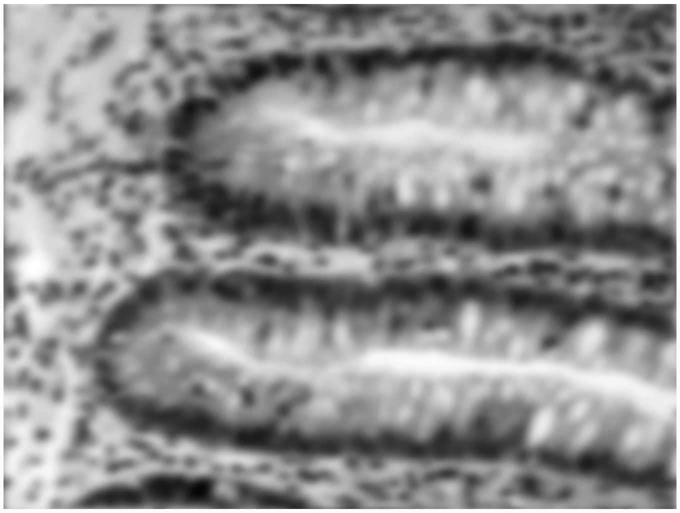

B

**FIGURE 10.** (A) The ratio image by selecting the largest value among the four ratios of black pixels covered by the respective moving windows to the total number of pixels in the windows; (B) Gaussian filtering; (C) the combined segmentation by summing up all the black pixels in the four images in Figs. 9 (A–D); and (F) thresholding segmentation of Gaussian filtering in (B).

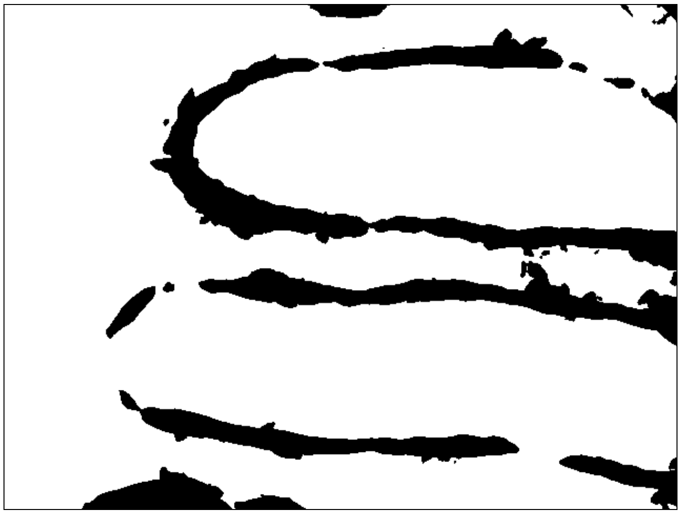

**FIGURE 10C**

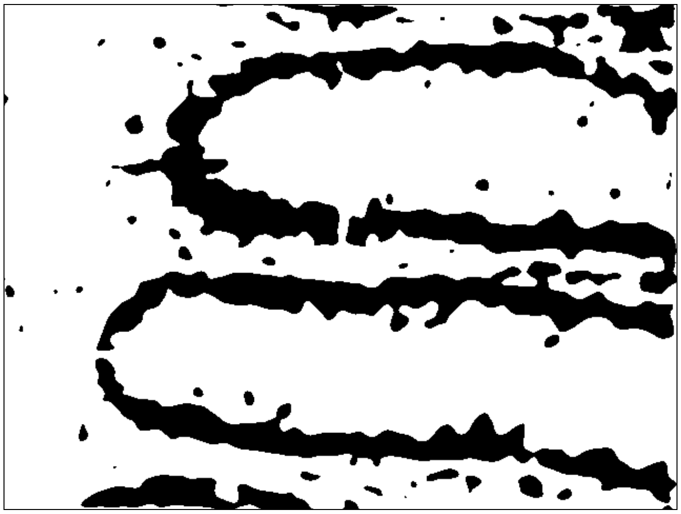

**FIGURE 10D**

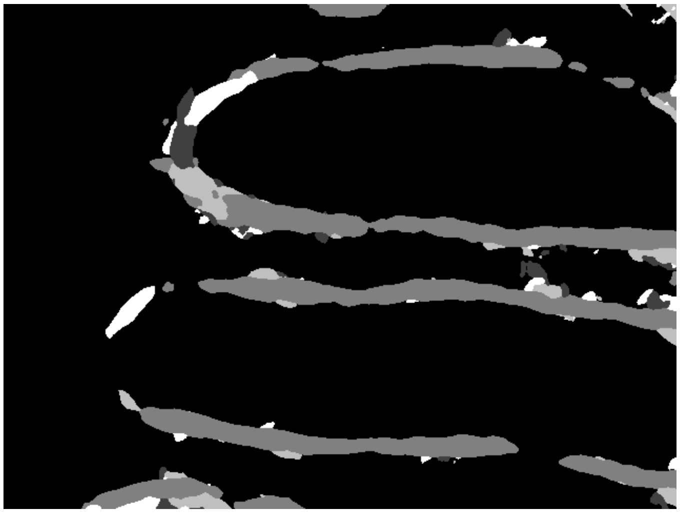

**A**

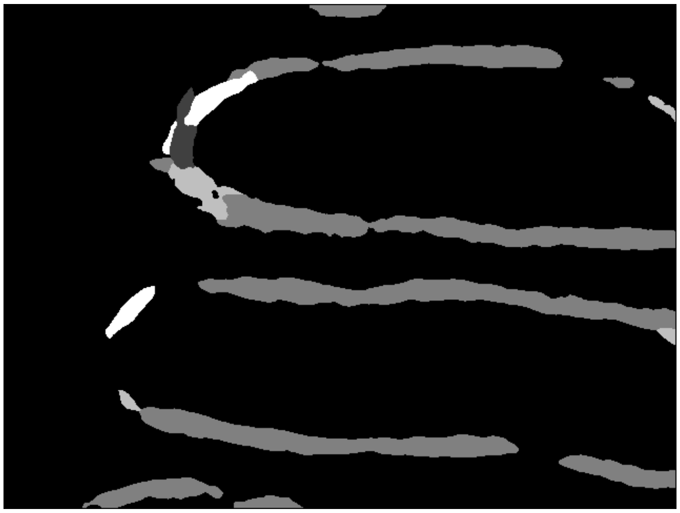

**B**

**FIGURE 11.** Postprocessing. (A) The mapping in which a nonblack level corresponds to the directional window that yields the largest ratio among the four; (B) defects either too small or unfit in shapes removed; (C) possible extension area with directional dilation; (D) expanding area; (E) final segmentation.

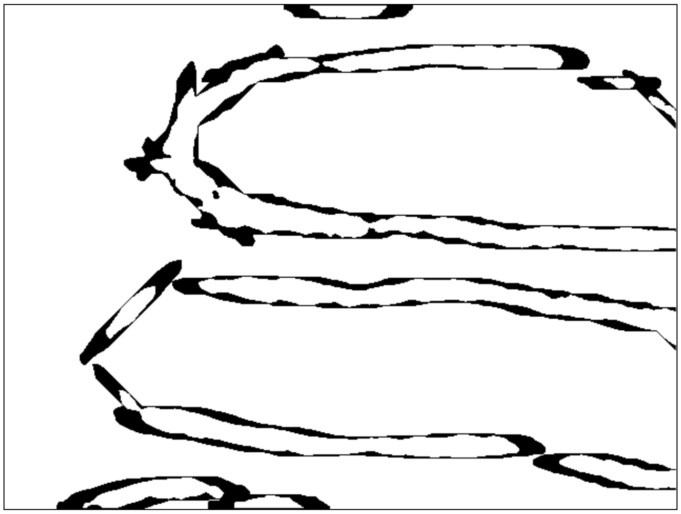

FIGURE 11C

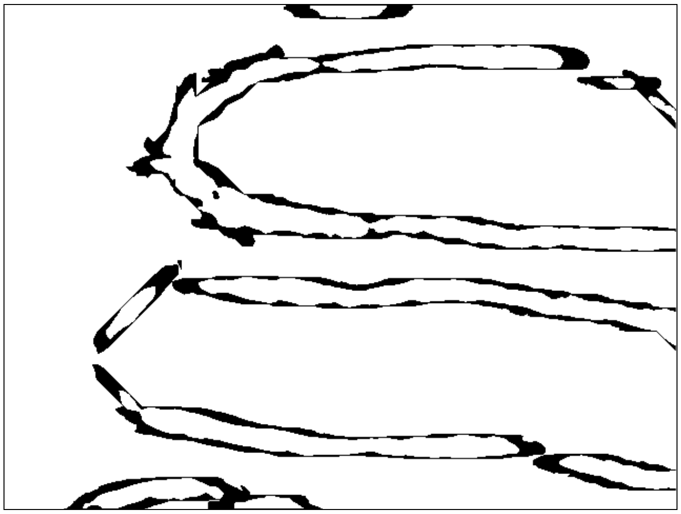

FIGURE 11D

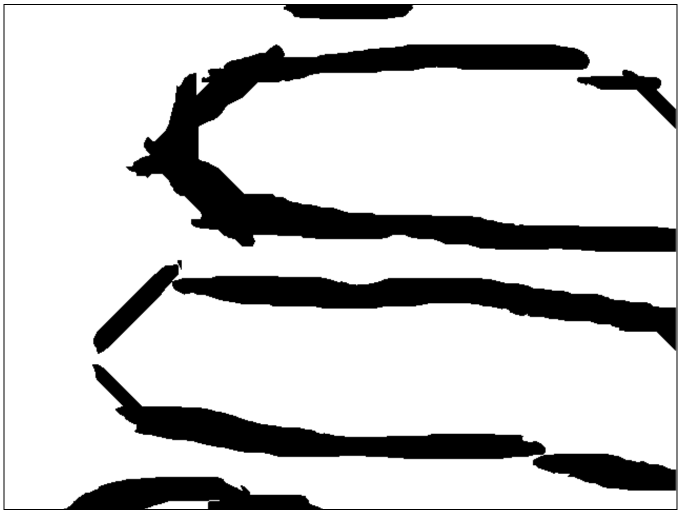

FIGURE 11E

## ACKNOWLEDGMENTS

We thank Dr. Anli Chen, Dr. Noam Harpaz, and Dr. Ruliang Xu, Mt. Sinai School of Medicine, for the materials, images and advices.
